# Assessment and Classification of Volatile Profiles in Melon Breeding Lines Using Headspace Solid-Phase Microextraction Coupled with Gas Chromatography-Mass Spectrometry

**DOI:** 10.3390/plants10102166

**Published:** 2021-10-13

**Authors:** Drishti Majithia, Rita Metrani, Nitin Dhowlaghar, Kevin M. Crosby, Bhimanagouda S. Patil

**Affiliations:** 1 Vegetable & Fruit Improvement Center, Department of Horticultural Sciences, Texas A&M University, 1500 Research Parkway, Suite A120, College Station, TX 77845, USA; drishtimajithia@tamu.edu (D.M.); ritametrani@tamu.edu (R.M.); nd288@tamu.edu (N.D.); k-crosby@tamu.edu (K.M.C.); 2 Department of Food Science & Technology, Texas A&M University, College Station, TX 77845, USA

**Keywords:** volatiles, melons, breeding lines, HS-SPME, GC-MS, flavor, aroma, principal component analysis (PCA)

## Abstract

*Cucumis melo* L is one of the most commercial and economical crops in the world with several health beneficial compounds as such carotenoids, amino acids, vitamin A and C, minerals, and dietary fiber. Evaluation of the volatile organic compounds (VOCs) in different melon (*Cucumis melo* L.) breeding lines provides useful information for improving fruit flavor, aroma, and antimicrobial levels. In this study, the VOCs in 28 melon breeding lines harvested in 2019 were identified and characterized using head space solid-phase microextraction (HS-SPME) coupled with gas chromatography-mass spectrometry (GC-MS). This identified 113 VOCs with significant differences in composition and contents of among the breeding lines, including 15 esters, 27 aldehydes, 35 alcohols, 14 ketones, 4 acids, 10 hydrocarbons, 5 sulfurs, and 3 other compounds. The highest average contents of all the VOCs were found in BL-30 (13,973.07 µg/kg FW) and the lowest were in BL-22 (3947.13 µg/kg FW). BL-9 had high levels of carotenoid-derived VOCs. The compounds with the highest contents were benzaldehyde, geranylacetone, and β-ionone. Quality parameters such as color and sugar contents of melons were also measured. All the melon color readings were within the typical acceptable range. BL-22 and BL-14 had the highest and lowest sugar contents, respectively. Principal component analysis (PCA) produced diverse clusters of breeding lines based on flavor and aroma. BL-4, BL-7, BL-12, BL-20, and BL-30 were thus selected as important breeding lines based on their organoleptic, antimicrobial, and health-beneficial properties.

## 1. Introduction

Muskmelons (*Cucumis melo* L.), including cantaloupe and honeydew melons, belong to the family Cucurbitaceae and are likely originated in Asia [1]. The United States (US) ranks first in the per capita consumption of melons as a fresh fruit [2]. The average American consumes ~13 kg of melon each year [3]. The most important melon traits associated with consumer preference are flavor, color, aroma, texture, juiciness, phytonutrients, and sweetness [4,5,6].

Sweetness and phytonutrient contents of melons are important quality attributes. Glucose, sucrose, and fructose make up the total sugar content (~97%) of melons. Sucrose accumulates as the major sugar (~50%) during ripening, with lower levels of fructose and glucose [7]. Muskmelons also have a large number of bioactive compounds such as folic acid, vitamin A (β-carotene), vitamin C (ascorbic acid), L-citrulline (a non-essential amino acid), and micronutrient elements such as iron, magnesium, and potassium [5,8]. These phytonutrients have numerous health-promoting properties such as anti-inflammatory, analgesic, antioxidant, anticancer, diuretic, antimicrobial, and antidiabetic activities [9,10,11]. Therefore, melon breeders have focused on producing improved melon cultivars with high sugar levels and enhanced phytonutrient contents.

In addition to sugar and phytonutrients, horticultural breeding programs have focused on long shelf life and improved food safety, especially for melons [12]. Indeed, the 2011 cantaloupe-related foodborne illness outbreak caused by Listeria monocytogenes was the deadliest outbreak in recent US history and caused a 32% reduction in the production of melons in the past decade. In addition, 43 outbreaks were associated with cantaloupe between 1998 and 2018 [13,14,15,16]. Plant volatile organic compounds (VOCs) have antimicrobial properties and help protect fruits against decay microorganisms [17]. Phytochemicals, including a wide range of VOCs, contribute to flavor and have been used as natural alternatives to improve the shelf life and safety of food [18]. Melon breeding programs aim to enhance the sustainability and profitability of melon production in the US by concentrating on consumer preferences and industry-driven needs. Therefore, exploration of melon VOC profiles provides key information for breeding programs aimed at producing safe, high-quality melons.

Ongoing research on melon VOCs has identified around 291 volatile compounds [19]. The key contributors to the total VOCs are esters, aldehydes, and ketones, along with smaller quantities of alcohols, sesquiterpenes, and sulfur-containing compounds [20,21,22]. These research programs have used various analytical techniques to identify and measure VOCs, including dynamic headspace extraction [23], stir bar sorptive technique [24], and solid phase microextraction (SPME) [25,26]. Head space (HS) [27] is a simple, solvent-free method for extraction and concentration of volatile compounds, combined with gas chromatography-mass spectrometry (GC-MS). We used this technique to analyze the VOCs in various citrus fruits [28,29].

Although several studies have evaluated the VOCs from melon cultivars [26,30,31], our knowledge of the VOC profiles in breeding lines of melon remains limited. Therefore, this study aimed to (a) characterize the VOCs from 28 melon breeding lines, (b) improve our knowledge about the aroma profile and VOC composition of various breeding lines, and (c) elucidate the relationship between volatile compounds and flavor, antimicrobial properties, and sugars. To the best of our knowledge, this is the first report pertaining to elucidation of volatiles from different melon breeding lines.

## 2. Results

### 2.1. Color Measurement

Consumers judge a food product by its color, taste, and aroma; therefore, the color of fresh produce is the one of the most important factors for consumer preferences [32]. The color attribute values of the cantaloupe fruit from different breeding lines are shown in Table 1. The L* (lightness) values ranged from 53.43 ± 8.03 to 73.9 ± 0.76; the a* (green to red) values were all in the positive range (15.39 ± 1.5 to 27.09 ± 1.1) showing that the melons tended towards a reddish hue due to the presence of carotenoids. A similar pattern was observed for b* (blue to yellow) values, where all the values were positive, ranging from 34.83 ± 5.32 to 49.06 ± 1.1 due to the presence of β-carotene [33].

### 2.2. Sugar Analysis

One of the major quality determinants in melon fruits is its sugar content, and sucrose is the most abundant sugar in melons (~97%), followed by fructose and glucose [7]. The contents of the individual sugars along with total sugar content are shown in Table 2. Sucrose contents ranged from 2.68–28.09 mg/g with the highest in BL-4 (28.09 mg/g) followed by BL-24 (26.88 mg/g). The fructose and glucose contents ranged from 4.42–24.01 mg/g and 3.39–11.33 mg/g, respectively, and BL-17 had the highest contents of these sugars. The total sugars ranged 19.17–55.54 mg/g, with the highest content in BL-22 followed by BL-14 (Table 2). The breeding lines BL-36 and BL-100 (10) were not available in enough quantities for their sugars to be measured adequately and therefore were not evaluated for sugar analysis.

### 2.3. Identification and Quantification of Volatile Compounds

The HS-SPME-GC-MS analysis identified a wide range of VOCs from the 28 melon breeding lines (Table 3). The 113 VOCs detected in the 28 melon lines included 27 aldehydes, 15 esters, 35 alcohols, 14 ketones, 10 hydrocarbons, 4 acids, and 5 sulfur-containing VOCs (Table 3).

**Figure 1:**
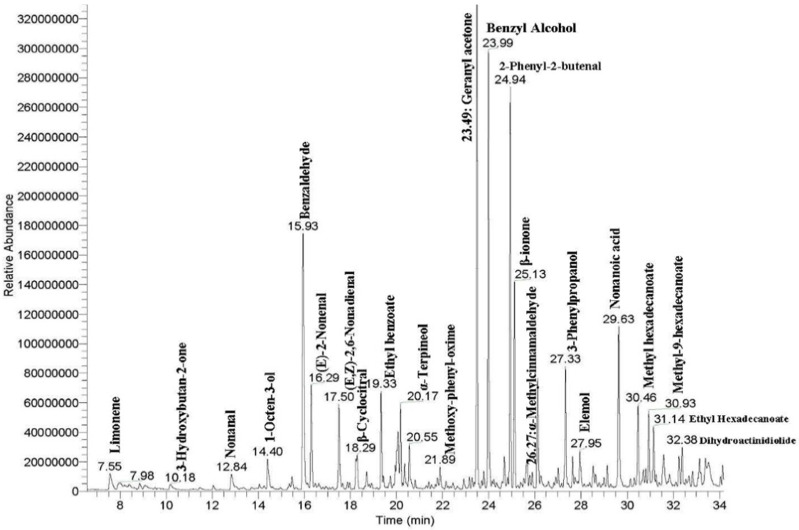
GC-MS profile of the volatile fraction of melon breeding line BL-20, using HS-SPME/GC-MS.

The other 3 volatiles identified were benzeneacetonitrile, 2-pentylfuran, and methoxy-phenyl-oxime. A typical chromatograph representing the melon VOCs in BL-20 is shown in Figure 1.

**Figure 2:**
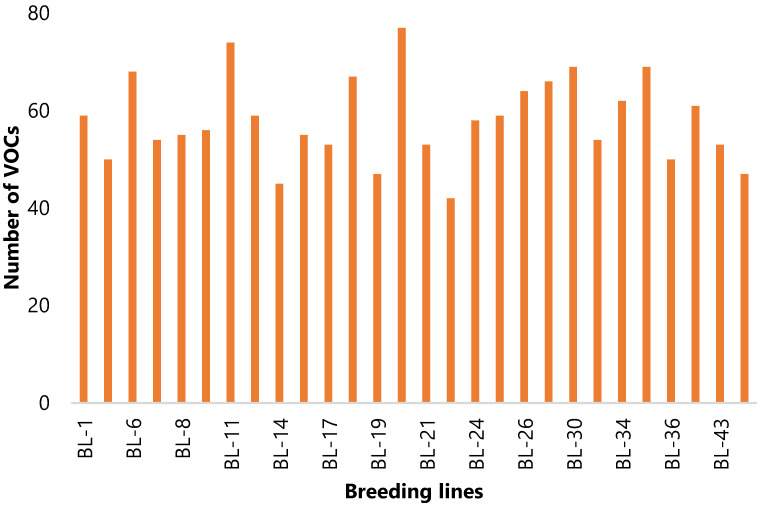
The representative of total number of individual volatile compounds present in each of the 28 melon breeding lines. Experiments were repeated individually thrice.

The highest total VOC content was found in BL-30 (13,973.07 µg/kg FW), whereas the lowest was found in BL-22 (3947.13 µg/kg FW); BL-20 had the most different VOCs (77) and BL-22 had the fewest different VOCs (42) (Table S1, Figure 2).

#### 2.3.1. Esters

Esters are major aroma compounds responsible for fruity notes and flavor characteristics in many fruits and vegetables [26,34]. Moreover, previous studies have demonstrated that climacteric varieties of melons appear to have high amounts of esters, whereas non-climacteric varieties lack esters and have comparatively low amounts of total aroma compounds [30,34,35,36]. Our breeding lines were all climacteric and, consistent with that, we identified fifteen esters in the cantaloupe breeding lines, with the total average volatile content of 3.78% in the 28 lines (Table S5). BL-26 (1227.84 µg/kg FW) had the highest total average content whereas BL-22 (22.74 µg/kg FW) had the lowest (Table S4). The ester present in the highest average content was ethyl benzoate (96.42 µg/kg FW) in BL-28 and BL-21. BL-26 and BL-28 showed high ethyl hexanoate levels (Table S3). In this study, ethyl hexadecanoate was identified in all the lines (Table S2). Ethyl hexadecanoate is known for its waxy flavor and aroma while ethyl benzoate and ethyl hexanoate are known for their fruity and musty aroma coupled with a minty flavor [37,38].

#### 2.3.2. Aldehydes

The 27 aldehydes identified here made up 37.16% of the total VOC composition (Table S5). The highest aldehyde content was found in BL-12 (6167.67 µg/kg FW) (Table S4), followed by BL-4 (5966.35 µg/kg FW); these lines exhibited high benzaldehyde contents (Table S3). Benzaldehyde, (*E*)-2-nonenal, and (*E*,*Z*)-2,6-nonadienal were present in all the lines (Table S3). BL-34 had the highest (*E*,*Z*)-2,6-nonadienal and (*E*)-2-nonenal contents (2028.31 µg/kg and 1039.43 µg/kg). Benzaldehyde is associated with an almond-like odor and flavor [39], whereas (*E*)-2-nonenal and (*E*,*Z*)-2,6-nonadienal exhibit strong waxy and cucumber-like flavors, respectively [40].

#### 2.3.3. Alcohols

Thirty-five alcohol compounds, contributing to 10.40–49.96% of the total VOC content were found in the lines (Table S5). High alcohol contents were found in BL-30 (6981.21 µg/kg FW) followed by BL-26 (3566.96 µg/kg FW) (Table S4). Benzyl alcohol (499.32 µg/kg FW) and 3-phenylpropanol (271.79 µg/kg FW) were the most abundant alcohol VOCs (Table S3), indicating their importance in melon aroma. Indeed, (*E,Z*)-3,6-nonadien-1-ol and benzyl alcohol are known to be major constituents of melon aroma [31]. BL-26 had the highest contents of benzyl alcohol and 3-phenylpropanol was the highest in BL-30. The lowest benzyl alcohol and 3-phenylpropanol content was observed in BL-22 (Table S3). Moreover, benzyl alcohol, 3-phenylpropanol, and α-terpineol were present in all the lines (Tables S2 and S3). A high level of α-terpineol (>260 µg/kg FW), was present in BL-30 and BL-7 (Table S3). Similarly, (*E*,*Z*)-3,6-nonadien-1-ol (>400 µg/kg FW) was found in BL-36 and BL-40.

#### 2.3.4. Ketones

Fourteen ketones, which accounted for 13.84–44.96% of the total VOC content, were identified (Table S5). BL-26 had the highest content (4583.84 µg/kg FW) of all ketones followed by BL-9 (4463.78 µg/kg FW), whereas BL-33 showed lowest total ketones (976.60 µg/kg FW) (Table S4). Geranylacetone and β-ionone showed the highest average contents (806.53 and 652.17 µg/kg FW), and were higher in BL-26 and BL-9 than in the other lines (Table S3). Geranylacetone and β-ionone are known for their fruity, tropical aroma and flavor [41]. Neryl acetone, an isomer of geranylacetone, has not been previously reported in melons, to the best of our knowledge. β-ionone, 6-methyl-5-hepten-2-one, β-ionone-epoxide, dihydroactinidiolide, and farnesyl acetone were the five ketones present in the BL (Table S2).

#### 2.3.5. Acids

In the present study, we identified four acidic VOCs: acetic acid, hexanoic acid, octanoic acid, and nonanoic acid, which accounted for an average of 0.94% of the total VOC contents in six of the lines (Table S5). BL-6 had the highest level (443.50 µg/kg FW) of acids and BL-19 had the lowest (5.10 µg/kg FW) (Table S4). Nonanoic acid showed the highest average content (40.29 µg/kg FW) followed by octanoic acid (21.12 µg/kg FW); BL-20 had high nonanoic acid contents and BL-6 had high octanoic acid contents (Table S3).

Short and medium-chain fatty acids such as hexanoic (C6:0) and octanoic (C8:0) acids have a wide spectrum of antimicrobial effects against bacteria, viruses, and fungi [42,43]. The present levels were in accordance with a previous study [44], which reported the fatty acids hexanoic acid (1.81%), octanoic acid (0.99%), and nonanoic acid (1.82%) in melons. Octanoic acid is naturally present in milk, coconut oil, and fruits and vegetables [45,46], and has generally been recognized as safe (GRAS) status [47]. Octanoic acid has a significant antimicrobial effect against *L. monocytogenes*, *Salmonella* spp., and *Escherichia coli* O157:H7 on various fresh produce such as spinach, grape tomatoes, and cantaloupe rind [48,49,50,51]. Moreover, fatty acids that are naturally present on tomato surfaces inhibit the growth of *Salmonella enterica* [52]. It is possible that increasing the levels of these acids in selected melons, along with other quality parameters would be useful for consumer safety.

#### 2.3.6. Hydrocarbons

An average of 1.18% of the total VOC content represented ten hydrocarbon compounds (Table S5). BL-35 had the highest content of hydrocarbons (206.39 µg/kg FW) among all the lines, and BL-43 had the lowest (41.41 µg/kg FW) (Table S4). Benzothiazole and α-calacorene were present in all the lines with 26.17 and 20.52 µg/kg FW average contents, respectively (Table S2); the highest benzothiazole level was identified in BL-4 (51.46 µg/kg FW) and the lowest in BL-24 (13.52 µg/kg FW). Moreover, the highest α-calacorene level was found in BL-36 (132.19 µg/kg FW) and the lowest was observed in BL-40 (7.45 µg/kg FW) (Table S3). While these compounds do not have particularly good aromatic properties, benzothiazole has anti-tumor and anti-microbial properties [53].

The hydrocarbon D-limonene was found in all the lines (70–75%) except BL-24, BL-26, BL-30, and BL-43. The highest D-limonene concentration was found in BL-20 (57.27 µg/kg FW). Limonene has significant health beneficial properties such as an anti-proliferative effect, prevention of gastric diseases, and anticancer activity, along with a lemony aroma [54,55,56,57]. Furthermore, D-limonene is a potent antimicrobial compound that inhibits the growth of foodborne pathogens such as *L. monocytogenes* and *Salmonella* spp. [58,59,60].

#### 2.3.7. Sulfurs

Five sulfur-containing VOCs were identified in this study. BL-28 showed the highest contents (516.20 µg/kg FW) and BL-4 had the lowest (4.25 µg/kg FW) (Table S3). Ethyl (methylthio)acetate was present in the highest average content (25.07 µg/kg FW), and has a major effect on the musky note of melon aroma [61,62] (Table S3).

#### 2.3.8. Others

Benzeneacetonitrile, 2-pentylfuran, and methoxy-phenyl-oxime made up 0.76% of the total VOCs (Table S5). To the best of our knowledge, methoxy-phenyl-oxime has only been previously reported in one other published study on melon [63]. Based on information from The Good Scents Company, benzeneacetonitrile and 2-pentylfuran have good aromatic properties. BL-35 had the highest total average content (113.58 µg/kg FW), whereas BL-8 had the lowest average content.

### 2.4. VOCs with Antimicrobial Properties in Selected Lines

The use of VOCs to extend the shelf life of food products has received substantial attention in recent years as an alternative to chemical preservatives [64,65]. One of the major objectives of our project was to enable the production of safer melons along with enhanced fruit and nutritional quality, flavor, and stress resistance. Several melon lines produced using genomics-assisted breeding were screened for the aforementioned attributes, including the 28 breeding lines reported in this study. Of note, our ongoing study indicates some of these lines have high levels of antimicrobials such as D-limonene, nonanoic acid, benzaldehyde, geranylacetone, and α-terpineol, which may reduce the overall persistence of foodborne pathogens such as *L. monocytogenes* and *Salmonella*.

In addition, many of the lines in this study produce fruit with smooth netted surfaces. As observed in our previous study [66], lightly netted rind surfaces have lower levels of microbial attachment. As also observed by Vitha [67], *Salmonella* showed a high attachment strength on densely netted cantaloupe rinds, followed by medium-netted and lightly netted rinds. Other factors such as attachment, transfer rates, dust, and contamination routes from contact surfaces also affect bacterial levels and susceptibility to the antimicrobials present on the rind surface [68]. Therefore, the VOCs identified in our melon breeding lines will be studied as potential biomarkers for their association with bacteria levels to improve food safety. The VOCs identified in specific lines and their antimicrobial efficacy are briefly discussed below.

BL-30 had a high average level of VOCs such as geranylacetone and 3-phenylpropanol, which contribute to the flavor and aromatic properties of various fruits [41]. Geranylacetone was also isolated from the horsetail *Equisetum arvense* L. and its essential oil form inhibited *Salmonella* [69]. Analogs of geranylacetone were tested for antimicrobial activities against *Staphylococcus aureus*, *Enterococcus*, *E. coli*, and *Klebsiella* spp. [70,71]. BL-30 had a high content of α-terpineol, which is usually found in citrus, tea tree, beer, and coffee, and is known for its antimicrobial properties against *E. coli* [72], *L. monocytogenes*, *Staphylococcus aureus*, and *Bacillus cereus* [64,73].

BL-20 had the highest number of total volatiles along with a high benzaldehyde content (1628.10 µg/kg FW). Benzaldehyde exhibits a strong antimicrobial effect against *L*. *monocytogenes* under anaerobic conditions [74]. The highest antimicrobial activity of benzaldehyde with surface sulfhydryl groups was on *Salmonella* followed by *Listeria* and *Lactobacillus* [75]. Benzaldehyde in the form of essential oil at 8–10 µL/mL, and as a polymer, inhibited foodborne pathogens in different fruit juices and broth [76,77]. BL-20 also had the highest contents of linalool (4.66 µg/kg FW), a compound known for its antimicrobial effect against *Salmonella* spp., *L. monocytogenes, E. coli* and *S. aureus* [78,79].

Nonanoic acid was the highest in BL-20 (349.88 µg/kg FW). Nonanoic acid is well known in the food industry for its antimicrobial and antifungal properties. It is used as a textile coating and inhibits the growth *of E. coli* and *S. aureus* [80]. Nonanoic acid, in an emulsion form, also proved to be more effective than traditional sanitizers against *Salmonella* growth on tomatoes during post-harvest storage [81].

BL-7 had high sucrose and total sugar contents, and had the highest contents of eucalyptol (34.49 µg/kg FW), which is known for its antimicrobial effect against *E*. *coli* and *S*. *aureus* [82]. BL-4 had high sucrose contents and was rich in β-ionone, α-terpineol, geranylacetone, benzaldehyde, and D-limonene. BL-12 had the highest benzaldehyde contents (4795.74 µg/kg FW), a high sucrose content, and high levels of good aromatic and flavorful compounds such as geranylacetone and β-ionone.

The breeding lines BL-30, BL-20, BL-7, BL-4, and BL-12, as described above, have good sensory properties, compounds with good antimicrobial activities, as well as high amounts of sugars. Additionally, BL-9 had a high a* value (Table 3) along with high levels of β-ionone (1568.57 µg/kg FW), dihydroactinidiolide (342.77 µg/kg FW), β-cyclocitral (308.21 µg/kg FW), α-ionone (22.84 µg/kg FW), and 2,6-dimethyl-5-heptenal (5.02 µg/kg FW); these compounds are associated with high lycopene and β-carotene contents in watermelons [83] and produce an orange hue, thus making the fruit visually appealing to the consumers. Carotenoids are also known to be beneficial to human health, as consuming a carotenoid-rich diet can reduce the risk of cancer, cardiovascular diseases, macular degeneration, cataracts, and UV-induced skin damage [84,85].

### 2.5. Principal Component Analysis (PCA)

**Figure 3:**
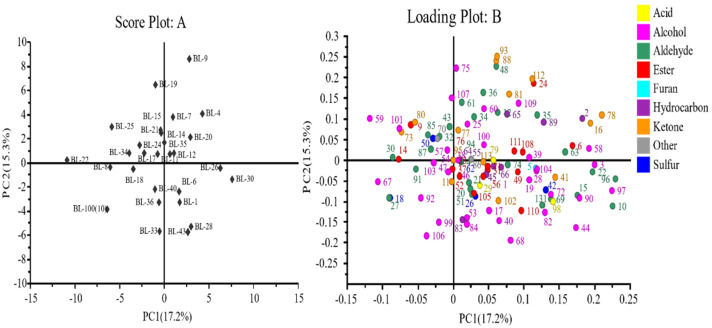
Principal component analysis of 28 melon breeding lines displayed according to their chemical classes. (**A**) corresponds to the scores plot. (**B**) corresponds to the loadings plot and the codes indicate the volatile compounds, as displayed in Table 3.

Principal component analysis (PCA) was used as a multivariate tool to detect correlations between all the breeding lines and VOCs. PC1 and PC2 accounted for 17.2% and 15.3% of the total variation, respectively (Figure 3). In the loadings plot (Figure 3B), most of the alcohols, aldehydes, and esters are on the right side of the PC2 axis, making up the 1st and 4th quadrants. The breeding lines correlating to these are BL-9, BL-7, BL-4, BL-20, BL-35, BL-12, BL-6, BL-1, BL-43, BL-28, BL-26, and BL-30. Since aldehydes, alcohols, and esters are the chemical classes usually associated with melon aroma and flavor [21,22], this suggests that these lines produce fruit with good odor and flavor properties. Some of the major compounds present in these quadrants are eucalyptol, benzaldehyde, carveol, citral, geranylacetone, limonene, cinnamaldehyde, α-calacorene, benzothiazole, β-ionone, and benzyl alcohol. However, the 4th quadrant of the loadings plot (Figure 3B) indicates the presence of two sulfurous and four acidic compounds, which suggests an intricate relationship between sensory properties and breeding lines.

Considering the 2nd and 3rd quadrants, which lie on the left side of the PC2 axis (Figure 3), we found that only a few volatile compounds associate with the breeding lines present in these two quadrants (BL-19, BL-25, BL-22, BL-34, BL-24, BL-17, BL-14, BL-21, BL100 (10), BL-8, BL-18, BL-40, BL-36, and BL-33). Methoxy-phenyl-oxime was associated with these lines; this compound is known to have anti-bacterial properties [86] and has been reported to be present in bamboo shoots [37,38], but has only been reported in muskmelons once [63]. Further research on its sensory and/or antibacterial properties should therefore be conducted.

Among all the breeding lines, the highest average VOC content was observed in BL-30 (13,973.07 µg/kg FW) (Table S1). 3-Phenylpropanol (1823.52 µg/kg FW), geranylacetone (1191.69 µg/kg FW), and 3-decen-1-ol (1070.61 µg/kg FW) were found in the highest quantities in BL-30 (average content > 1000 µg/kg FW) (Table S3). 3-Phenylpropanal is known for its fruity, spicy, and floral aroma [87], and geranylacetone exhibits a fruity, tropical aroma and flavor and is also known for its anti-microbial properties [70]. The highest number of volatiles was observed in BL-20 (77), with benzaldehyde (1628.10 µg/kg FW) followed by geranylacetone (1223.77 µg/kg FW) exhibiting the highest average contents (Table S3).

The lines characterized here had more aldehydes and alcohols than esters, which implies that the melons were not fully mature, since the presence of esters is mostly associated with ripe, mature melons whereas aldehydes are mostly associated with immature fruits [35]. This is in accordance with the color readings (Table 2), where BL-4, BL-7, BL-11, BL-15, BL-28, BL-30, BL-33, BL-34, and BL-40 were associated with lightness and green color along with high contents of aldehydes and alcohols (Figure 3A,B).

**Figure 4:**
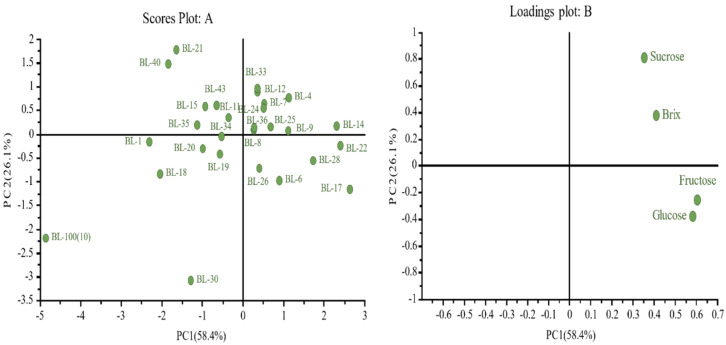
Principal component analysis displaying the breeding lines correlation with individual and total sugars. (**A**) corresponds to the breeding lines and loadings plot; (**B**) corresponds to the three sugars and total sugar.

BL-33, BL-7, BL-4, BL-24, BL-36, BL-9, BL-14, BL-25, BL-26, BL-6, BL-28, and BL-22 were positively associated with the three main sugars along with total sugars (Figure 4). Although BL-17 and BL-12 did not have total sugar values associated to them, BL-17 was positively correlated to glucose and fructose, while BL-12 had more sucrose. Future breeding programs could focus on selecting lines with good sensory properties, i.e., high aroma and high sugar content, and with compounds having anti-microbial and health beneficial properties.

## 3. Materials and Methods

### 3.1. Plant Materials

**Figure 5:**
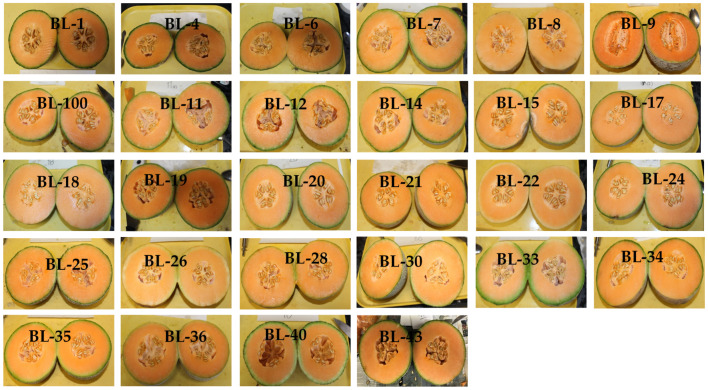
Images of melons from the 28 breeding lines harvested in 2019.

In this study, 28 melon breeding lines were cultivated in the year 2019 at the Texas AgriLife Research and Extension Center, Weslaco, Texas between March and July (Figure 5). All the fruits were transported to the Vegetable and Fruit Improvement Center (VFIC), College Station, Texas, for further analysis.

### 3.2. Chemicals and Reagents

Gas chromatographic and HPLC results were verified using authentic standards including (*R*)-(+)-limonene, 2-pentylfuran, 6-methylhept-5-en-2-one, ethyl heptanoate, 1-hexanol, dimethyl trisulfide, (*Z*)-3-hexan-1-ol, ethyl caprylate, 1-octen-3-ol, decanal, benzaldehyde, ethyl butyrate, (*E*)-2-heptenal, (*E*,*Z*)-2,6-nonadienal, (*E*)-carveol, geranylacetone, β-ionone, benzothiazole, thymol, eucalyptol, 2-methyl-1-butanol, (*E*)-2-hexenal, ethyl hexanoate, octanal, 1-octen-3-ol, nonanal, (*E*)-2-octenal, ethyl (methylthio)acetate, ethyl (methylthio)acetate, 2,6-dimethyl-5-heptenal, hexyl acetate, 2-ethyl-2-hexenal, ethyl hexadecanoate, citronellal, benzyl alcohol, (*E*,*E*)-2,4-ethylhexadienoate, (*E*,*E*)-2,4-heptadienal, decanal, 1-octanol, ethyl-3 (methylthio)propionate, (*E*)-2-nonenal, 2,3-butanediol, β-cyclocitral, phenylacetaldehyde, ethyl decanoate, ethyl benzoate, 1-chlorodecane, 1-nonanol, α-terpineol, 1-decanol, α-farnesene, ethyl phenylacetate, α-isomethyl-ionone, α-ionone, β-ionol, 2-phenyl-2-butenal, 1-dodecanol, cinnamaldehyde, 3-phenylpropanol, octanoic acid, nonanoic acid, farnesylacetone, nootkatone (internal standard), and *n*-alkane standards (C_5_–C_24_). SPME fibers, glucose, sucrose, and fructose were obtained from Sigma-Aldrich (St. Louis, MO, USA). Sodium chloride (Fisher Scientific, Pittsburg, PA USA) was added to improve the extraction of volatile compounds.

### 3.3. Color Analysis

The color of the fruit samples was measured using a Minolta CR-400 Chroma Meter (Konica Minolta Sensing, Inc., Osaka, Japan). The instrument was first calibrated using a white tile standard calibration plate (Calibration Plate CR-A43, Minolta Cameras, Osaka, Japan). In brief, 30mL of juice was transferred into the liquid tester of colorimeter following which the measurements for L* (0, black to 100, white), a* (−60, green to +60, red), and b* (−60, blue to +60, yellow) were taken for all the samples in triplicates. In the CIELAB color space, the L* indicates lightness, a* indicates the greenness-redness axis with −a* representing green and +a* representing red, and b* indicates the blueness-yellowness axis with −b* representing blue and +b* being yellow.

### 3.4. Sugar Sample Preparation and Measurement Using HPLC

Five grams of the pureed sample was placed in a 50 mL centrifuge tube and 5 mL of nanopure water from the NANOPure system (Barnstead/Thermolyne, Dubuque, IA, USA) was added. The sample mixture was then homogenized at 7000 rpm (850 Homogenizer, Thermo Fisher Scientific, Waltham, MA, USA) for 30 s and then subjected to sonication for 30 min. The tubes were then centrifuged at 10,000× *g* for 15 min after which the supernatant was transferred into 15 mL tubes. Then, 900 µL of the decanted solution was mixed with 300 µL of methanol in microfuge tubes and centrifuged again (8000× *g* for 5 min) to yield a clear solution. Each breeding line sample was prepared in triplicate and the final mixture was stored at −20 °C until analysis.

For analysis, 20 µL of the processed sample was injected into the HPLC system consisting of a binary pump, autosampler, refractive index detector (Perkin Elmer LC 200 Series, Norwalk, CT, USA), and Reze × RCM-Monosaccharide Ca^+2^ (300 mm × 7.8 mm) column with a guard column Carbo-Ca (4 mm × 3 mm ID) (Phenomenex, Inc. Torrance, CA, USA). Nanopure water was used as mobile phase with a flow rate of 0.6 mL/min, while the column temperature was maintained at 80 °C. Standard curves for fructose, glucose, and sucrose were used for calculating the sugar contents.

### 3.5. Sample Preparation for HS-SPME-GC-MS

Cantaloupes were longitudinally cut into four halves; the seeds were then removed, and the flesh was separated from the rind using a knife. The flesh from each fruit was then cut into small cubes and blended in a high-speed blender (Oster, Milwaukee, WI, USA) for 1 min to form a puree. One gram of puree from each sample was then weighed into a 20 mL headspace vial, to which 1 mL NaCl (30%, *w*/*v*) and 5 µL of nootkatone (0.025%, *v*/*v*) was added as an internal standard. Each sample was prepared in triplicate and stored at −20 °C until GC-MS analysis.

### 3.6. HS-SPME-GC-MS Analysis

Melon samples were kept at room temperature for 30 min and then loaded into a TriPlus RSH auto-sampler (Austin, TX, USA). The volatile compounds were extracted using HS-SPME with a 50/30 µm carboxen/polydimethylsiloxane/divinylbenzene (CAR/PDMS/DVB) fiber. The extraction and the desorption time using SPME fibers were 30 min and 2 min respectively, at 80 °C, with constant agitation for 10 s every 2 min. Following adsorption, the SPME fiber was injected into the GC injector at 225 °C. Helium gas was used as a carrier gas with a constant flow rate of 1 mL/min in splitless mode. Volatile analysis of the samples was performed using the Thermo Finnegan gas chromatogram coupled with Dual-Stage Quadrupole (DSQII) mass spectrometer (Thermo Fisher Scientific, Inc., San Jose, CA, USA; Thermo Fisher, Austin, TX, USA). Restek Rtx-Wax column (30 m × 0.25 mm id with 0.25 µm film thickness; Restek Corp., Bellefonte, PA, USA) was used for analysis. The initial oven temperature was held at 40 °C for 2 min, and then increased to 210 °C at a rate of 5 °C/min, with a total run time of 37 min. The MS detector operated in the electronic ionization mode (70 eV), in a scan mode from 30 to 300 amu at a rate of 11.5 scans/sec. The mass transfer line and ion source temperature were maintained at 280 and 285 °C, respectively.

An additional positive ionization step was carried out with methane as a reagent gas at a flow rate of 1 mL/min. The mass transfer line and ion source temperatures were 230 °C and 180 °C, respectively.

### 3.7. Identification and Quantification of Volatile Compounds

The data were processed using Xcalibur software (v. 2.0.7, Thermo Fisher Scientific, Inc, San Jose, CA, USA). Volatile compounds were identified by comparing their Kovats indices (KI), mass spectra, and retention times to their respective standards. KI values were calculated under the same conditions as the samples, namely by calculating the retention times of *n-*alkane standards (C_5_–C_24_). Identification of the VOCs was based on comparing the sample mass spectra with NIST 05 Mass Spectral Database (NIST, Gaithersburg, MD, USA) and Wiley 8 library. Nootkatone was used as an internal standard to perform the quantification of volatiles. The results were expressed as g/kg fresh weight of the sample.

### 3.8. Statistical Analysis

Analysis of each experiment was carried out in triplicate and all data were represented as the mean SD. The principal component analysis (PCA) was performed using the mean data with SIMCA 16.0.2 statistical software (Umetrics Inc., San Jose, CA, USA). All the data were normalized using log transformation to have a normal distribution.

## 4. Conclusions

This study showed that breeding lines differ greatly based on their individual volatile compositions (VOC contents and types), sugar contents, and color. BL-30 and BL-20 showed the highest average VOC content and the most different VOCs. Other important lines to be considered for future projects, based on their antimicrobial and health beneficial properties, are BL-12, BL-4, BL-7, and BL-9. In this study, we found one new compound- neryl acetone, which, to the best our knowledge, has not been previously reported in melons, and will be further evaluated for its sensory and antibacterial properties in our future projects. The three main volatiles found in abundance based on their average concentrations were benzaldehyde, geranylacetone, and β-ionone. D-limonene was present in all the breeding lines and has potential antimicrobial efficacy on foodborne pathogens. Moreover, 32 of the compounds identified here have antimicrobial properties with major compounds such as hexanal, octanoic acid, nonanoic acid, carveol, citral, cinnamaldehyde, α-calacorene, benzothiazole, β-ionone and benzyl alcohol -calacorene, benzothiazole, and benzyl alcohol. Our future studies include determining the invitro antimicrobial activities and the plants extract from cantaloupe against foodborne pathogens from these breeding lines. Our findings will be valuable for the development of healthier melons with high sugar contents and high levels of antimicrobial compounds.

## Figures and Tables

**Table 1:** Color attributes L* (lightness), b* (blue to yellow), a* (green to red) in melon breeding lines. Means with the same letter indicate no significant difference.

Name	L*	b*	a*
BL-1	73.90 ± 0.76a	46.58 ± 1.94abc	21.02 ± 0.89a
BL-4	59.94 ± 3.59abc	41.02 ± 2.28abcd	19.19 ± 1.21ab
BL-6	73.11 ± 11.74ab	39.21 ± 3.98bcd	20.56 ± 0.704c
BL-7	59.46 ± 8.00abc	39.29 ± 4.16bcd	17.66 ± 1.84ab
BL-8	73.57 ± 3.13a	45.07 ± 4.04abc	16.37 ± 1.68abc
BL-9	54.60 ± 5.37bc	40.70 ± 3.32abcd	23.30 ± 1.49a
BL-11	62.48 ± 7.54abc	40.73 ± 1.69abcd	17.75 ± 1.14ab
BL-12	62.76 ± 6.68abc	42.58 ± 2.73abcd	20.65 ± 1.45a
BL-14	70.56 ± 1.26abc	47.70 ± 0.79ab	20.86 ± 0.32a
BL-15	57.58 ± 2.69abc	41.26 ± 2.05abcd	20.53 ± 1.02a
BL-17	67.18 ± 6.27abc	45.54 ± 2.45abc	22.43 ± 1.46a
BL-18	63.03 ± 7.81abc	42.89 ± 4.73abcd	21.35 ± 1.93a
BL-19	67.26 ± 0.36abc	49.06 ± 1.10a	27.09 ± 1.10a
BL-20	72.96 ± 2.81ab	46.13 ± 2.89abc	20.48 ± 1.76a
BL-21	66.65 ± 8.37abc	44.88 ± 4.18abc	20.61 ± 1.75a
BL-22	68.14 ± 10.63abc	44.59 ± 6.42abc	18.71 ± 1.77ab
BL-24	66.06 ± 7.51abc	46.11 ± 3.40abc	22.01 ± 0.70a
BL-25	53.43 ± 8.03c	37.59 ± 4.61cd	20.87 ± 1.69a
BL-26	56.46 ± 2.11abc	40.37 ± 1.09abcd	17.04 ± 0.87abc
BL-28	64.05 ± 8.70abc	41.71 ± 5.70abcd	18.31 ± 1.53ab
BL-30	62.55 ± 10.87abc	41.22 ± 4.93abcd	20.12 ± 1.48ab
BL-33	68.97 ± 11.56abc	34.83 ± 5.32d	15.39 ± 1.50bc
BL-34	64.51 ± 8.02abc	40.48 ± 3.35abcd	17.07 ± 2.22abc
BL-35	56.97 ± 2.54abc	43.30 ± 2.49abcd	21.71 ± 1.99a
BL-36	60.63 ± 1.96abc	42.41 ± 2.70abcd	20.33 ± 2.41a
BL-40	55.16 ± 2.29abc	41.67 ± 2.64abcd	19.45 ± 0.75ab
BL-43	66.50 ± 3.45abc	44.09 ± 0.26abcd	20.24 ± 0.33a
BL-100 (10)	72.94 ± 0.56ab	48.44 ± 1.11ab	18.25 ± 1.38ab

**Table 2:** Sucrose, glucose, fructose, and total sugar concentration (mg/g) in melon breeding lines. Means with the same letter indicate no significant difference.

Name	Sucrose	Glucose	Fructose	Total Sugars
BL-1	10.10 ± 0.52cde	4.15 ± 0.81bcd	4.91 ± 1.14h	19.17 ± 0.75f
BL-4	28.09 ± 0.79a	5.97 ± 0.21abcd	16.10 ± 0.36abcdef	50.17 ± 1.25abc
BL-6	14.86 ± 10.55abcde	9.05 ± 2.87abcd	14.02 ± 1.94bcdefg	37.94 ± 4.59abcdef
BL-7	19.86 ± 0.88abcd	5.19 ± 1.51bcd	13.21 ± 1.97bcdefgh	38.27 ± 1.91abcdef
BL-8	17.45 ± 3.49abcde	6.59 ± 0.05abcd	10.03 ± 3.22defgh	34.07 ± 3.01bcdef
BL-9	21.14 ± 0.83abcd	7.42 ± 0.11abcd	14.36 ± 0.4bcdefg	42.93 ± 1.34abcde
BL-11	16.41 ± 5.03abcde	4.57 ± 1.03bcd	11.11 ± 1.98defgh	32.10 ± 3.65cdef
BL-12	23.61 ± 10.53abc	5.20 ± 1.32bcd	12.48 ± 3.73cdefgh	41.3 ± 7.28abcde
BL-14	25.43 ± 0.06abc	9.32 ± 0.04abc	18.93 ± 0.86abcd	53.69 ± 0.83ab
BL-15	14.23 ± 0.33abcde	3.59 ± 0.06cd	9.78 ± 0.03efgh	27.61 ± 0.42def
BL-17	15.40 ± 0.71abcde	11.33 ± 0.4a	24.01 ± 0.68a	50.75 ± 1.76abc
BL-18	7.55 ± 4.43de	3.49 ± 1.21cd	8.79 ± 2.6efgh	19.84 ± 4.17f
BL-19	12.18 ± 0.92bcde	4.77 ± 0.36bcd	12.21 ± 0.75cdefgh	29.17 ± 2.04def
BL-20	12.08 ± 10.73bcde	5.80 ± 1.85abcd	6.55 ± 0.14gh	24.43 ± 3.9ef
BL-21	19.34 ± 0.19abcd	3.43 ± 0.06cd	4.42 ± 0.15h	27.20 ± 0.28def
BL-22	23.67 ± 0.72abc	9.91 ± 0.22ab	21.94 ± 0.94ab	55.54 ± 1.87a
BL-24	26.88 ± 1.69ab	5.63 ± 0.25abcd	14.46 ± 0.76bcdefg	46.98 ± 2.67abcd
BL-25	20.14 ± 4.51abcd	6.17 ± 1.94abcd	14.55 ± 4.34bcdefg	40.87 ± 4.93abcde
BL-26	14.07 ± 0.38abcde	6.29 ± 0.13abcd	16.57 ± 0.44abcde	36.93 ± 0.95abcdef
BL-28	16.91 ± 11.22abcde	8.29 ± 5.09abcd	20.87 ± 8.75abc	46.08 ± 1.26abcd
BL-30	2.68 ± 2.11e	6.22 ± 2.96abcd	10.93 ± 1.95defgh	19.84 ± 2.00f
BL-33	20.55 ± 2.99abcd	4.71 ± 1.9bcd	11.99 ± 3.81cdefgh	37.27 ± 4.14abcdef
BL-34	10.65 ± 0.81cde	5.43 ± 1.28abcd	7.53 ± 2.05efgh	23.61 ± 1.43ef
BL-35	13.72 ± 2.9abcde	5.23 ± 1.19bcd	5.94 ± 1.4gh	24.90 ± 1.22ef
BL-40	18.98 ± 0.63abcd	3.39 ± 0.2d	4.81 ± 0.05h	27.20 ± 0.47def
BL-43	18.50 ± 0.77abcd	5.39 ± 0.16bcd	7.07 ± 0.13fgh	30.97 ± 1.06cdef

**Table 3:** Concentration ranges (mg/kg) of volatile compounds recovered from 28 melon breeding lines via HS-SPME GC-MS harvested in the year 2019. The volatile compounds were identified by comparing the mass spectra and Kovats indices (KI).

Code ^a^	RT ^b^	Compounds	Measured RI ^c^	Library RI ^g^	ID	Concentration Range (µg/kg)
1	7.55	Limonene	1178	1035	MS ^d^, RI	0–57.27
2	7.58	Eucalyptol	1180	1198	MS, RI	0–34.49
3	8.35	2-Methyl-1-butanol	1213	1206	MS, RI, STD ^e^	0–222.36
4	8.38	(*E*)-2-Hexenal	1214	1220	MS, RI	0–151.66
5	8.56	2-Pentylfuran	1221	1231	MS, RI, PICI ^f^	0–83.7
6	8.75	Ethyl hexanoate	1229	1234	MS, RI	0–548.93
7	9.08	*Trans*-α-ocimene	1242	1237	MS, RI	0–36.22
8	9.49	1-Pentanol	1258	1252	MS, RI	0–6.25
9	9.75	Hexyl acetate	1268	1275	MS, RI	0–182.4
10	10.19	Octanal	1285	1286	MS, RI, STD, PICI	0–320.98
11	10.19	3-Hydroxybutan-2-one	1285	1289	MS, RI, PICI	0–429.21
12	10.78	2,6-Dimethyl-5-heptenal	1308	1315	MS, RI	0–5.02
13	10.98	(*Z*)-2-Heptenal	1316	1323	MS, RI, PICI	0–24.34
14	11	(4*E*)-4-Hexenyl acetate	1317	1326	MS, RI	0–182.77
15	11.31	2-Ethyl-2-hexenal	1329	1330	MS, RI, PICI	0–333.96
16	11.45	6-Methyl-5-hepten-2-one	1334	1341	MS, RI	0–43.11
17	12.04	1-Hexanol	1357	1360	MS, RI	0–365.47
18	12.35	Dimethyl trisulfide	1369	1383	MS, RI, PICI	0–19.31
19	12.83	(*Z*)-3-Hexen-1-ol	1388	1386	MS, RI	0–366.45
20	12.84	Nonanal	1388	1396	MS, RI, STD, PICI	0–162.94
21	13	(2*E*,4*E*)-Hexadienal	1395	1400	MS, RI, PICI	0–41.47
22	13.7	(*E*)-2-Octenal	1421	1432	MS, RI, STD, PICI	3.45–94.91
23	13.95	(*E*)-4-Nonenal	1431	1435	MS, RI, PICI	0–9.54
24	13.95	Ethyl caprylate	1431	1440	MS, RI, PICI	0–95.43
25	14.04	3,7-Dimethyloctan-3-ol	1435	-	MS, RI	0–22.54
26	14.25	Ethyl (methylthio)acetate	1443	1452	MS, RI, STD	0–293.87
27	14.25	(*E*)-6-Nonenal	1443	1453	MS, RI, PICI	0–132.62
28	14.45	1-Octen-3-ol	1450	1456	MS, RI, PICI	0–231.06
29	14.45	Acetic acid	1450	1480	MS, RI	0–194.67
30	14.95	Citronellal	1469	1495	MS, RI	0–29.95
31	15.11	Ethyl 2,4-hexadienoate	1476	1501	MS, RI, STD	0–27.85
32	15.28	(*E*,*E*)-2,4-Heptadienal	1482	1506	MS, RI, STD, PICI	0–158.44
33	15.34	Methyl nonanoate	1484	1515	MS, RI	0–15.39
34	15.46	Decanal	1489	1521	MS, RI, PICI	22.45–62.45
35	15.95	Benzaldehyde	1508	1530	MS, RI, PICI	134.35–4795.74
36	16.3	(*E*)-2-Nonenal	1521	1543	MS, RI, PICI	60.09–1039.43
37	16.44	Ethyl nonanoate	1527	1548	MS, RI	0–26.49
38	16.67	2,3-Butanediol	1535	1550	MS, RI	0–17.28
39	16.79	Linalool	1540	1552	MS, RI	0–4.66
40	17.08	1-Octanol	1551	1561	MS, RI, STD	0–468.01
41	17.15	3,5-Octadien-2-one	1554	1567	MS, RI, PICI	0–113.59
42	17.11	Ethyl-3(methylthio)propionate	1552	1571	MS, RI, STD	0–112.05
43	17.5	(*E*,*Z*)-2,6-Nonadienal	1567	1596	MS, RI, PICI	31.89–2028.31
44	17.65	3-Octen-1-ol	1573	1563	MS, RI	0–453.78
45	17.85	Hexadecane	1581	1600	MS, RI	0–116.33
46	17.97	Terpinen-4-ol	1585	1612	MS, RI	0–12.98
47	17.97	Isopulegol	1585	1606	MS, RI	0–14.51
48	18.24	β-Cyclocitral	1596	1623	MS, RI, PICI	63.31–308.21
49	18.55	Octyl-2-methylbutanoate	1610	1634	MS, RI, PICI	0–16.34
50	18.55	3-(Methylthio)propyl acetate	1610	1633	MS, RI	0–70.41
51	18.7	Phenylacetaldehyde	1617	1640	MS, RI, STD, PICI	11.4–121.98
52	18.81	Ethyl decanoate	1622	1642	MS, RI, PICI	0–45.74
53	18.93	4-Methyl-5-decanol	1628	-	MS, RI, PICI	0–104.14
54	18.93	Isopinocarveol	1628	1642	MS, RI	0–6.01
55	19.15	β-Cedrene	1639	1648	MS, RI	0–3.41
56	19.33	Ethyl benzoate	1647	1650	MS, RI, PICI	0–607.17
57	19.43	1-Nonanol	1652	1655	MS, RI	0–32.84
58	19.66	cis-Verbenol	1663	1663	MS, RI	0–11.18
59	19.95	(*Z*)-3-Nonenol	1677	1682	MS, RI, PICI	13.02–514.19
60	20.17	α-Terpineol	1688	1688	MS, RI, STD	129.04–314.73
61	20.34	Dodecanal	1696	1710	MS, RI, PICI	18.45–93.15
62	20.67	3-(Methylthio)propanol	1712	1711	MS, RI, PICI	0–100.65
63	20.81	Citral	1719	1714	MS, RI, PICI	0–33.21
64	20.92	1,4-Dimethoxybenzene	1724	1728	MS, RI, PICI	0–6.04
65	21.2	α-Farnesene	1738	1747	MS, RI, STD, PICI	0–25.56
66	21.29	δ-Cadinene	1742	1748	MS, RI, PICI	0–15.81
67	21.39	(*E*,*Z*)-3,6-Nonadien-1-ol	1747	1749	MS, RI, PICI	0–416.92
68	21.69	1-Decanol	1761	1760	MS, RI, PICI	0–77.79
69	21.75	3-Phenylpropanal	1764	1783	MS, RI	0–62.85
70	21.88	Methoxy-phenyl-oxime	1770	-	MS, RI, PICI	21.7–84.66
71	22	Ethyl phenylacetate	1776	1786	MS, RI, STD	0–79.32
72	22.14	3-Decen-1-ol	1783	1790	MS, RI, PICI	0–1070.61
73	22.44	1-Phenyl-1,2-propanedione	1797	1818	MS, RI, PICI	0–155.53
74	22.47	(*E*,*E*)-2,4-Decadienal	1799	1826	MS, RI, PICI	0–40.46
75	23.16	Carveol	1833	1836	MS, RI, PICI	17.27–56.98
76	23.27	Ethyl dodecanoate	1839	1840	MS, RI	0–10.11
77	23.27	α-Isomethyl-ionone	1839	1848	MS, RI, STD, PICI	0–27.22
78	23.31	α-Ionone	1841	1849	MS, RI, STD, PICI	0–22.84
79	23.38	Hexanoic acid	1844	1854	MS, RI	0–51.87
80	23.48	Neryl acetone	1849	1865	MS, RI, PICI	0–1700.85
81	23.48	Geranylacetone	1849	1865	MS, RI, PICI	0–3152.62
82	23.98	Benzyl alcohol	1874	1880	MS, RI, PICI	13.75–1067.05
83	24.59	α-Calacorene	1904	1920	MS, RI, PICI	7.45–132.19
84	24.67	2-Phenylethanol	1908	1915	MS, RI, PICI	0–238.75
85	24.82	Tetradecanal	1915	1919	MS, RI, PICI	13.49–122.04
86	24.83	Benzeneacetonitrile	1916	1931	MS, RI, PICI	0–28.92
87	24.92	2-Phenyl-2-butenal	1920	1932	MS, RI, STD, PICI	0–549.66
88	25.12	β-Ionone	1930	1947	MS, RI, PICI	198.54–1568.57
89	25.39	Benzothiazole	1943	1948	MS, RI, PICI	13.52–51.46
90	25.54	β-Ionol	1950	1968	MS, RI	0–62.15
91	25.67	3-Phenyl-2-butenal	1957	-	MS, RI, PICI	0–125.5
92	25.9	1-Dodecanol	1969	1970	MS, RI, STD	0–133.89
93	26.15	β-Ionone epoxide	1981	1977	MS, RI, PICI	81.43–673.58
94	26.27	α-Methylcinnamaldehyde	1987	1992	MS, RI, PICI	0–26.87
95	26.82	γ-Nonalactone	2008	2018	MS, RI, PICI	0–141.3
96	27.02	Cinnamaldehyde	2013	2025	MS, RI, STD, PICI	0–305.08
97	27.32	3-Phenylpropanol	2021	2058	MS, RI, STD, PICI	4.59–1823.52
98	27.65	Octanoic acid	2030	2070	MS, RI, STD, PICI	0–165.72
99	27.81	Globulol	2034	2085	MS, RI	0–14.38
100	27.93	Elemol	2038	2090	MS, RI, PICI	61.95–120
101	28.63	Cedrenol	2057	2110	MS, RI, PICI	0–29.76
102	29	γ-Decalactone	2067	2144	MS, RI, PICI	0–236.55
103	29.53	Eugenol	2081	2162	MS, RI, PICI	0–21.77
104	29.58	*T*-Cadinol	2082	2165	MS, RI	0–57.1
105	29.62	Nonanoic acid	2083	2169	MS, RI, STD, PICI	0–349.88
106	29.77	1-Tetradecanol	2087	2174	MS, RI, PICI	0–319.76
107	30.12	δ-Cadinol	2097	2179	MS, RI	15.53–31.85
108	30.46	Methyl hexadecanoate	2127	2202	MS, RI, PICI	0–125.98
109	30.67	α-Cadinol	2152	2217	MS, RI	0–72.28
110	30.93	Methyl 9-hexadecanoate	2182	2278	MS, RI, PICI	0–275.22
111	31.12	Ethyl hexadecanoate	2204	2288	MS, RI, PICI	13.76–299.48
112	32.38	Dihydroactinidiolide	2352	2291	MS, RI, PICI	45.89–342.77
113	33.12	Farnesyl acetone	2439	2363	MS, RI, PICI	17.89–156.17

^a^ Compound codes. ^b^ Retention time in minutes. ^c^ Retention indices measured relative to *n*-alkanes (C_5_–C_24_). ^d^ MS: Mass spectra. ^e^ STD: Standard comparison, compounds identified using authentic standards. ^f^ PICI: Positive ionization chemical ionization; compounds identified using positive ionization-chemical ionization mode. ^g^ Kovats retention indices values reported in the database obtained from the NIST library (https://www.nist.gov, accessed on 6 October 2021).

## Supplementary Materials

The following are available online at https://www.mdpi.com/article/10.3390/plants10102166/s1, Table S1: Number of volatiles present and total content of volatiles present in each BL; Table S2: No of BLs present in each volatile compound; Table S3: Relative contents (µg/kg FW) of each type of compound class in 28 Breeding lines; Table S4: Total content (µg/kg) of each type of volatiles in 28 melon breeding lines; Table S5: Total % of each type of volatiles in 28 melon breeding lines.
